# Comparative efficacy of corticosteroid injection and non-invasive treatments for plantar fasciitis: a systematic review and meta-analysis

**DOI:** 10.1038/s41598-018-22402-w

**Published:** 2018-03-05

**Authors:** Chien-Min Chen, Meng Lee, Chia-Hung Lin, Chia-Hao Chang, Chu-Hsu Lin

**Affiliations:** 1 Department of Physical Medicine and Rehabilitation, Chang Gung Memorial Hospital, Chiayi, Taiwan; 2 School of Medicine, College of Medicine, Chang Gung University, Taoyuan, Taiwan; 3 Department of Neurology, Chang Gung Memorial Hospital, Chiayi, Taiwan; 4 Department of Nursing, Chang Gung University of Science and Technology, Chiayi Campus, Chiayi, Taiwan

## Abstract

The first choice of treatment for patients with plantar fasciitis is non-invasive treatment, rather than corticosteroid injection (CSI). However, no comprehensive study has compared the effectiveness of CSI with non-invasive treatments for plantar fasciitis. We conducted a meta-analysis comparing CSI and non-invasive treatment effects on plantar fasciitis. The primary outcome was pain reduction. Nine randomized controlled trials comparing CSI with 4 non-invasive treatment types were included. A trend favoring CSI over non-invasive treatments was indicated regarding reduction in the visual analogue scale (VAS) score at 1–1.5 (mean difference (MD), 1.70; 95% confidence interval (CI) = 0.39–3.01; *P* = 0.01) and 2–3 months (MD, 1.67; 95% CI = 0.58–2.76; *P* = 0.003). At 1.5-month follow-up, CSI was associated with improved VAS score compared with physical therapy (PT) (MD, 2.5; 95% CI = 0.1–4.9; *P* = 0.04). No significant differences in the VAS score reduction were observed between CSI and shock wave therapy within 3 months. In summary, CSI tends to be more effective for pain reduction than non-invasive treatments within 3 months. Moreover, CSI provides significant pain relief at 1.5 months after treatment compared with PT. This study provides important clinical information for selecting therapeutics.

## Introduction

Plantar fasciitis is the most common cause of inferior heel pain^1^. Typical symptoms include pain with the first weight-bearing step in the morning, and the diagnosis can be made clinically. Some clinicians prefer to use the term plantar fasciopathy in lieu of plantar fasciitis to de-emphasise the inflammatory component because pathological examination of the fascia typically reveals degeneration over inflammation^2^.

Although the cause of plantar fasciitis can be multifactorial, the most common etiology is biomechanical stress of the plantar fascia at its enthesis of the calcaneal tuberosity^3^. Prolonged weight-bearing, obesity, limited ankle joint dorsiflexion, posterior muscle group tightness and maladaptive patterns of walking or running can produce biomechanical stress of the plantar fascia^2^.

To date, there are various treatments for plantar fasciitis. Common treatments can be divided into non-invasive treatments, such as physical therapy (PT)^4–6^, orthosis^7,8^, oral nonsteroidal anti-inflammatory drugs (NSAIDs)^9^, radiation therapy (RT)^10^ and shock wave (SW)^11^ and invasive treatments, such as corticosteroid injection (CSI)^12,13^, botulinum toxin injection^14^, platelet-rich plasma (PRP) injection^15^ and surgery^16^. PT for plantar fascia comprises stretching exercises and mobilization to improve lower extremity joint mobility and flexibility of the plantar fascia^5^. Therapeutic ultrasound, a type of PT modality, is applied to the posterior heel to increase tissue circulation and metabolism, and to soften the plantar fascia^6^. Orthosis includes splinting, enhancing the mechanical control of the foot and ankle^7^ or providing insoles for shock absorption along the inferior foot^8^. Oral NSAIDs are known to inhibit cyclooxygenase-2, thereby exerting an anti-inflammatory effect on the plantar fascia to relieve pain^9^. RT treatments expose the heel area to radiation to reduce inflammation over the treated area^10^. SW treatment applies high pulse SW energy to the insertion zone of the plantar fascia, potentially healing the degenerative tissue of the plantar fascia^11^.

Corticosteroid can accelerate the process of pain relief through its strong anti-inflammatory effect. The mechanism of action of corticosteroid, which is administered via injection, involves the inhibition of fibroblast proliferation and ground substance protein expression, which are observed in the pathological features of plantar fasciitis^13^. Botulinum toxin, which is injected in the plantar fascia and gastrocnemius-soleus muscular complex, can reduce the tension in the plantar fascia and relax the muscles^14^. PRP stimulates the natural healing process by promoting platelet growth factors, thereby accelerating the physiological healing process of the plantar fascia^15^. Surgical procedures, such as fasciotomy for plantar fascia release^16^, are usually reserved as last-resort options for treating plantar fasciitis in patients with persistent recalcitrant heel pain after receiving other treatments.

A meta-analysis found that CSI provided better pain relief than placebo in treating plantar fasciitis^17^. Compared to botulinum toxin and PRP injection, CSI is cheaper, more easily prepared and performed and has an acceptable therapeutic effect. There has been no evidence showing that botulinum toxin and PRP injection provided a better therapeutic effect than CSI^18^. Currently, CSI is still one of the first-line treatments for plantar fasciitis^19^. Nevertheless, from the viewpoint of patients with plantar fasciitis, if non-invasive treatments could provide a therapeutic effect equal to that of CSI, these patients would choose non-invasive treatments over CSI as their first choice. However, a review of the literature yielded no comprehensive studies comparing the efficacy of CSI with that of non-invasive treatments for plantar fasciitis. Therefore, the present study aimed to conduct a meta-analysis that compared the therapeutic efficacy of CSI and that of non-invasive treatments for plantar fasciitis.

## Methods

This study was conducted in accordance with the recommendations of the Preferred Reporting Items for Systematic Reviews and Meta-Analysis (PRISMA) Statement^20^.

### Data sources and searches

We searched the PubMed, EMBASE, MEDLINE, Cochrane Central Register of Controlled Trials, Scopus and Web of Science databases using the following terms: plantar fasciitis, plantar fasciopathy, corticosteroid, steroid and glucocorticoid in the title or abstract from the earliest records up to 22 June 2017. There were no language restrictions.

### Study selection

The inclusion criteria of the present study were as follows: (1) a study that compared the efficacy of CSI and a non-invasive treatment for patients with plantar fasciitis or plantar fasciopathy, with a non-invasive treatment being defined as one not penetrating the body by incision or injection; (2) a randomized controlled trial (RCT); and (3) a full-text article reporting a pain score, e.g., visual analogue scale (VAS), both before and after the treatments, as well as a pain reduction score (the change value in pain score from before to after treatments) that could be obtained. The VAS, directly reflecting subjective pain experienced at a particular body part^21^, has been widely used to quantify heel pain^12,22^. The VAS is self-reported, with 0 denoting no pain and 10 denoting the worst possible pain. The exclusion criteria of the present study were as follows: (1) the reporting of studies that combined another treatment with either CSI or non-invasive treatment during the entire treatment period; (2) the article was a case report, review, study protocol, or abstract; or (3) no pain score was reported. Two investigators (C.M.C. and M.L.) developed the selection criteria, and one of them (C.M.C.) conducted the literature search. Another investigator (C.H.L.) assessed these criteria and independently checked the enrolled trials. Discrepancies were resolved by discussion with a third investigator (C.H.L.) and by referencing the original report.

### Data extraction and quality assessment

All data from eligible studies were extracted by 2 independent investigators according to a standard protocol. The extracted data included patient characteristics, treatment regimens, timing of treatment administration and details of outcome measurements. The quality of each RCT was evaluated using the Jadad scale^23^, in which the aggregate score ranged from 0 to 5 points.

### Data synthesis and analysis

Data were extracted from baseline measurements and at 1–1.5 and 2–3 months after the initial treatment. The mean change in pain reduction score from baseline is the primary outcome. Continuous variables were compared using the mean difference (MD) and accompanying 95% confidence intervals (CIs).

Where significant heterogeneity existed (*I*^2^ ≥ 50%), a random-effects analysis was applied. The Cochrane Collaboration’s Review Manager software package (RevMan 5.3) was used for this analysis. A *P*-value of <0.05 was considered statistically significant.

## Results

### Characteristics of included studies

**Figure 1:**
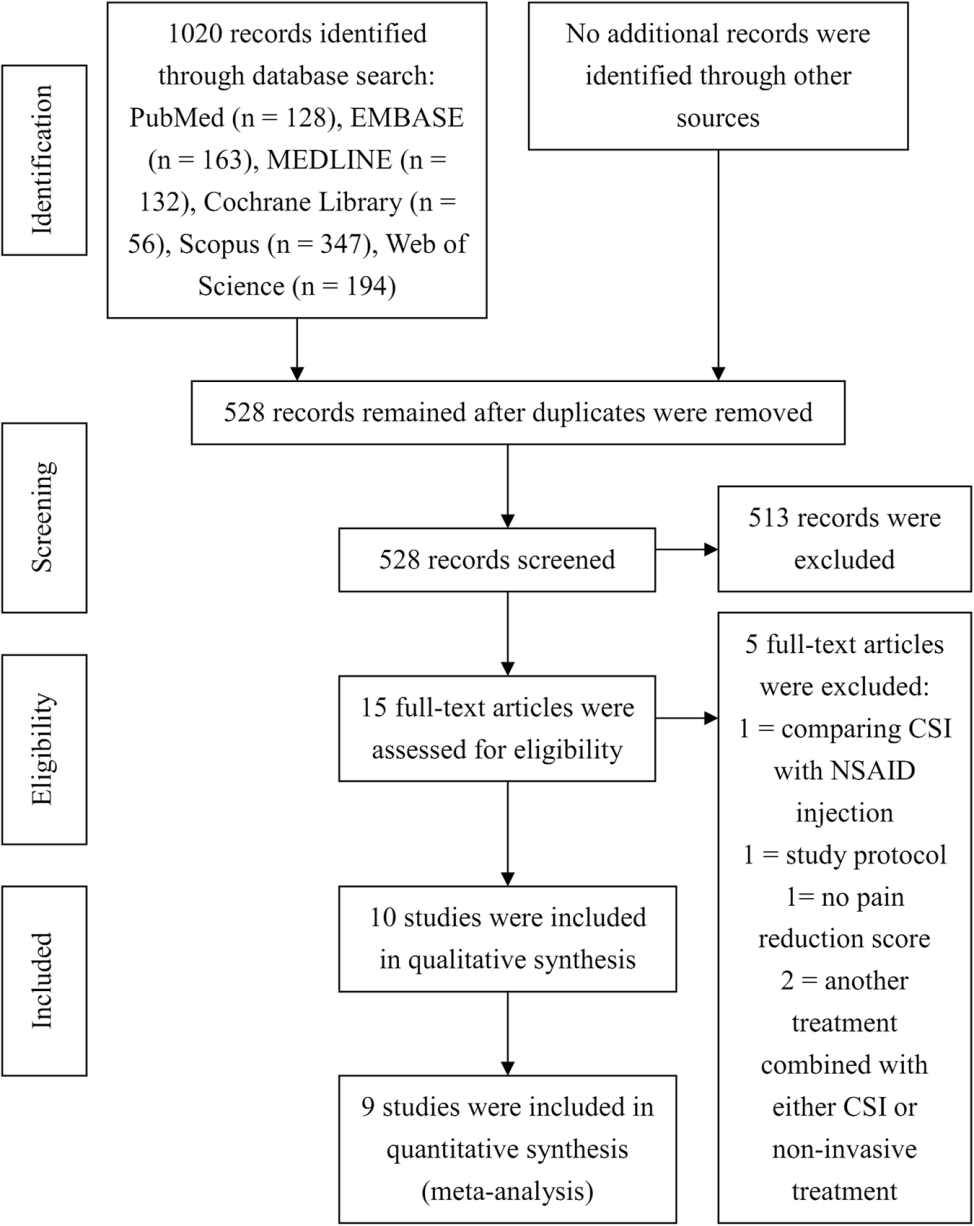
Flow diagram of the included studies.

At the end of the article selection process, 10 RCTs were included in the qualitative synthesis (Table 1). Of these, 1 RCT compared CSI with RT but lacked standard deviation values, which are necessary for a meta-analysis. Therefore, 9 RCTs were ultimately selected for the meta-analysis. Figure 1 shows the screening and study selection procedure. Of the 9 RCTs, 5 described CSI vs SW, 2 described CSI vs PT, 1 described CSI vs insole and 1 described CSI vs NSAIDs. CSI was administered as a single injection in all 9 trials. SWs were administered 1–5 times, and the energy efflux intensity was 0.03–0.2 mJ/mm^2^ in 5 trials. PT was individually administered 11 times for a 3-week duration and 84 times for a 12-week duration in 2 trials. Insole was used daily for 1 month. For NSAIDs, patients were given 1 oral tablet of diclofenac (50 mg) twice a day for 4 weeks.

**Table 1:** Characteristics of the included studies.

Reference	Year	Study type	n/n(CSI)/n(the other group)	Average age, years	Average BMI, kg/m^2^	Average disease duration, months	Treatment cycles	Details of interventions	Outcome measures	Follow-up, months
CSI vs SW										
Eslamian *et al*.^11^	2016	RCT	40/20/20	42.9 vs 41.5	NR	2.6 vs 2.1	1 for CSI; 5 for SW	CSI, 40 mg methylprednisolone + 1 mL of 1% lidocaine SW, 2000 pulses, 0.2 mJ/mm^2^, 15 min per session, five sessions at 3-day intervals	VAS, FFI	0, 1, 2
Mardani-Kivi *et al*.^32^	2015	RCT	84/41/43	44.7 vs 43.9	29.1 vs 30.2	<1.5	1 for CSI; 3 for SW	CSI, 1 mL of 40 mg methyl prednisolone acetate + 1 mL of 2% lidocaine SW, 2000 pulses, 0.15 mJ/mm^2^, once a week for 3 weeks	VAS	0, 0.75, 1.5, 3
Porter *et al*.^30^	2005	RCT	125/64/61	39.9 vs 38.6	NR	3.7 vs 3.2	1 for CSI; 3 for SW	CSI, 1 mL of 5.7 mg betamethasone + 2 mL of 1% lignocaine SW, 1000 pulses, 0.08 mJ/mm^2^, 3 times at weekly intervals	VAS, TT	0, 3, 12
Sorrentino *et al*.^34^	2008	RCT	60/30/30	NR	NR	at least 2	1 for CSI; 4 for SW	CSI, 1 mL of 40 mg methylprednisolone + 0.6 mL of 3% mepivacaine hydrochloride, ultrasound-guided SW, 2000 pulses, 0.03 mJ/mm^2^, 4 sessions at weekly intervals	VAS, PFT	0, 1.5
Yucel *et al*.^35^	2010	RCT	60/33/27	44.7 vs 42.9	NR	9.9 vs 9.4	1 for CSI; 1 for SW	CSI, 0.5 mL of combined 6.43 mg/mL betamethasone dipropionate and 2.63 mg/mL betamethasone sodium phosphate + 0.5 mL of 2% prilocaine hydrochloride SW, 3000 pulses, >0.12 mJ/mm^2^ for one time	VAS, HTI, TRR	0, 3
CSI vs PT										
Celik *et al*.^5^	2016	RCT	39/20/19	45.6 vs 45.4	30.6 vs 29.4	13.1 vs 11.2	1 for CSI; 11 for PT	CSI, antiseptic solution + 1 mL of 40 mg methylprednisolone acetate + 4 mL of 2% prilocaine hydrochloride PT, subtalar traction, talocrural dorsal glide, subtalar lateral glide, first tarsometatarsal joint dorsal glide, gastrocnemius stretching, plantar fascia-specific stretching, for 9 visits and twice at home during a 3-week period	VAS, FAAM	0, 0.75, 1.5, 3, 12
Ryan *et al*.^4^	2014	RCT	56/28/28	46.2 vs 52.4	26.2 vs 24.3	71.4 vs 69.4	1 for CSI; 84 for PT	CSI, 1 mL of dexamethasone + 0.5 mL of 1% lidocaine + daily calf-stretching programme PT, karaoke, balance walking, forefoot extension exercise, standing one-legged balance exercise, ankle inversion/eversion exercise, gastrocnemius and soleus stretching, tissue-specific plantar fascia stretch, daily for a 12-week period	VAS, PFT, FADI, FFAA, LFAA	0, 1.5, 3
CSI vs Insole										
Yucel *et al*.^8^	2013	RCT	40/20/20	45.6 vs 47.4	30.8 vs 29.3	6.8 vs 7.8	1 for CSI; 30 for insole	CSI, 1 mL of 6.43 mg/mL betamethasone dipropionate and 2.63 mg/mL betamethasone sodium phosphate combination + 1 mL of 20 mg/2 mL lidocaine HCl, ultrasound-guided Insole, full-length silicone insole, daily for 1 month	VAS, PFT, FAOS, HTI,	0, 1
CSI vs NSAIDs										
Biswas *et al*.^9^	2011	RCT	120/60/60	41.7 vs 38.4	NR	<3	1 for CSI; 56 for NSAIDs	CSI, 1 mL of 40 mg methylprednisolone + 2 mL of 0.5% bupivacaine NSAIDs, one oral tablet diclofenac (50 mg) twice a day for 4 weeks	VAS	0, 0.25, 0.5, 1, 2
CSI vs RT										
Canyilmaz *et al*.^10^	2015	RCT	124/64/60	54.7 vs 52.6	33.1 vs 34	14 vs 18.6	1 for CSI; 6 for radiation	CSI, 1 mL of 40 mg methylprednisolone + 0.5 mL of 1% lidocaine Radiation, total dose of 6.0 Gy applied in 6 fractions of 1.0 Gy three times a week	VAS, FLFS, MPPS	0, 3, 6

CSI = corticosteroid injection; SW = shock wave; PT = physical therapy; NSAIDs = nonsteroidal anti-inflammatory drugs; RT = radiation therapy; RCT = randomized controlled trial; NR = not reported; BMI = body mass index; VAS = visual analogue scale; FFI = foot function index; TT = tenderness threshold; PFT = plantar fascia thickness; HTI = heel tenderness index; TRR = therapeutic response rate; FAAM = foot and ankle ability measure; FADI = foot and ankle disability index; FFAA = frequency focal anechoic areas; LFAA = length of focal anechoic area; FAOS = foot and ankle outcome score; FLFS = five-level function score; MPPS = modified von Pannewitz pain score.

### Methodological assessment

The quality of the included studies is shown in Table 2. Of the the 9 RCTs included in the meta-analysis, 4 had a Jadad score of <3 and 5 (2 CSI vs SW, 2 CSI vs PT and 1 CSI vs insole) had a score of ≥3.

**Table 2:** Jadad scales of reporting randomized controlled trials for meta-analysis.

Reference	Year	Randomization is mentioned	Appropriateness of randomization	Blinding is mentioned	Appropriateness of blinding	An account of all patients	Total
CSI vs SW							
Eslamian *et al*.	2016	1	1	1	0	1	4
Mardani-Kivi *et al*.	2015	1	0	0	0	1	2
Porter *et al*.	2005	1	1	0	0	1	3
Sorrentino *et al*.	2008	1	1	0	0	0	2
Yucel *et al*.	2010	1	0	0	0	1	2
CSI vs PT							
Celik *et al*.	2016	1	1	1	0	1	4
Ryan *et al*.	2014	1	1	0	0	1	3
CSI vs Insole							
Yucel *et al*.	2013	1	1	0	0	1	3
CSI vs NSAIDs							
Biswas *et al*.	2011	1	0	0	0	1	2

CSI = corticosteroid injection; SW = shock wave; PT = physical therapy; NSAIDs = nonsteroidal anti-inflammatory drugs.

### Outcome measurement

**Figure 2:**
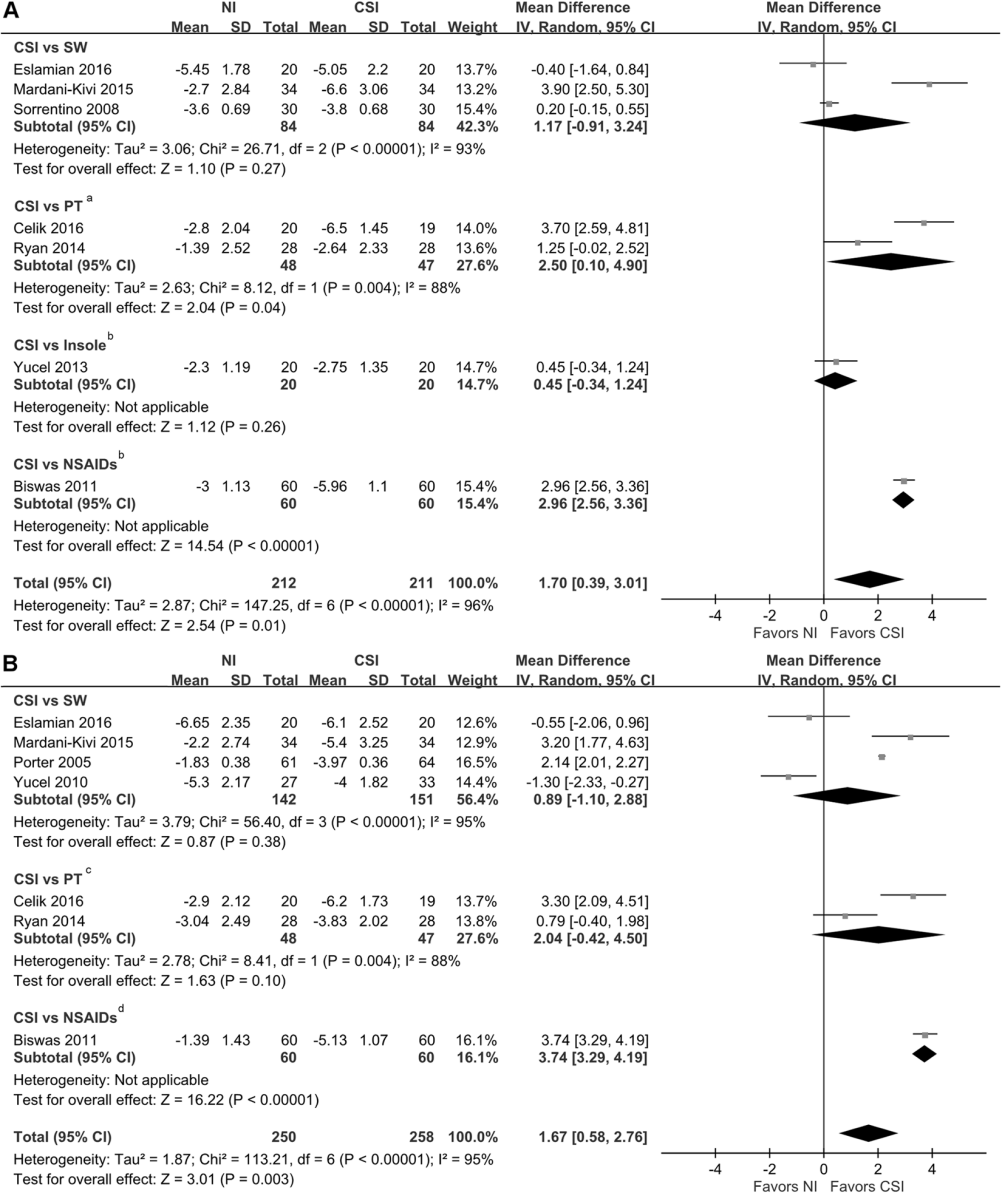
Forest plot of comparisons of pain reduction between corticosteroid injection and non-invasive treatments. (**A**) VAS score reduction at 1–1.5 months. (**B**) VAS score reduction at 2–3 months. ^a^Indicates the follow-up time point was 1.5 months. ^b^Indicates the follow-up time point was 1 month. ^c^Indicates the follow-up time point was 3 months. ^d^Indicates the follow-up time point was 2 months. NI = non-invasive.

The present study reported outcomes using VAS. Figure 2A shows the differences between CSI and non-invasive treatments and the between-group differences in VAS score reduction at 1–1.5 months. VAS score reduction was significant between CSI and non-invasive treatments at 1–1.5-month follow-up (MD, 1.70; 95% CI = 0.39–3.01; *P* = 0.01). VAS score reduction was significantly greater in the CSI group than in the PT group at 1.5-month follow-up (MD, 2.5; 95% CI = 0.1–4.9; *P* = 0.04). VAS score reduction was significantly greater in the CSI group than in the NSAIDs group at 1-month follow-up (MD, 2.96; 95% CI = 2.56–3.36; *P* < 0.01; heterogeneity, not applicable for a single trial). No significant differences were observed in VAS score reduction between CSI and SW at 1–1.5-month follow-up (MD, 1.17; 95% CI = –0.91–3.24; *P* = 0.27).

The differences between CSI and non-invasive treatments and the between-group differences in VAS score reduction at 2–3 months are shown in Fig. 2B. VAS score reduction at 2–3 months was significant between CSI and non-invasive treatments (MD, 1.67; 95% CI = 0.58.–2.76; *P* = 0.003). The difference in VAS score reduction between CSI and NSAID groups at 2 months was significant (MD, 3.74; 95% CI = 3.29–4.19; *P* < 0.01; heterogeneity, not applicable for a single trial). No significant differences in VAS score reduction between CSI and SW at 2–3-month follow-up were found (MD, 0.89; 95% CI = −1.1–2.88; *P* = 0.38).

## Discussion

The present meta-analysis compared the efficacy of CSI and that of non-invasive treatments in the treatment of plantar fasciitis. The major finding of the present study is that CSI resulted in better pain relief from plantar fasciitis than non-invasive treatments at 1–1.5 and 2–3 months after treatment. Another major finding is that CSI had better therapeutic effect in pain relief than PT at 1.5 months after treatment. Although the present study indicated that CSI had better result than did PT in terms of pain reduction at 3 months, no significant differences were observed between CSI and PT at 3 months. In the study by Tsai *et al*.^22^, the VAS score in patients who received CSI for plantar fasciitis decreased from 5.46 (pre-injection) to 1.50 (2 weeks post-injection) and 0.77 (2 months post-injection) and then went up to 2.46 (1 year post-injection). Another study^12^ found that, among patients receiving CSI for plantar fasciitis, VAS scores decreased from 6.44 (pre-injection) to 3.38 (3 weeks post-injection) and then ticked up slightly to 3.63 (3 months post-injection). The time of maximum therapeutic effect with CSI might be <3 months post-injection. We speculate this is the reason why the effect of CSI was not more significant than that of PT at 3 months post-treatment.

To the best of our knowledge, there have been only a few articles published that discuss the effect of PT on plantar fasciitis. The studies by Celik *et al*.^5^ and Ryan *et al*.^4^ were the 2 that compared the efficacy of CSI and PT for the treatment of plantar fasciitis in our present study. The PT interventions in the 2 studies were stretching exercises, and participants were individually instructed by a trained therapist initially and the therapist provided follow-ups and patient guidance in sessions thereafter. Celik *et al*.’s article^5^ illustrated that patients with plantar fasciitis received 11 times of stretching exercises and joint mobilization for 3 weeks, and significant improvement in pain reduction was detected not only at 3 months but also at 1 year after treatment. We considered the possibility that the effect could last up to 1 year because these patients were advised to repeat the same stretching exercises on their own; however, completion the these self-stretching exercises was not documented. Given the lack of sufficient evidence of the therapeutic effectiveness of PT for plantar fasciitis, more studies focusing on the outcomes, times and types of PT for plantar fasciitis should be conducted in the future.

Currently, plantar fasciitis is considered not only an inflammatory condition but also a degenerative process^24,25^. Theoretically, SW could increase the proliferation of growth factors, peripheral blood circulation, angiogenesis and neovascularization in the degenerative tissue of the heel^26^, and SW is therefore believed to be effective in the treatment of plantar fasciitis. Moreover, several meta-analyses have documented the advantages of SW over placebo treatments^27,28^. Our present meta-analysis yielded no differences in the effectiveness of SW and CSI at 1–1.5 and 2–3 months after treatment. A study^29^ by Hsiao *et al*. also found similar results. Hsiao *et al*.^29^ reported a better therapeutic effect in CSI than in SW at 3 months after treatment; however, it also did not reach significant difference. Studies comparing SW with CSI for longer follow-up periods, such as 6 months or 1 year, are scarce. An RCT conducted by Porter *et al*.^30^, found that both SW and CSI to be effective in reducing pain in treating plantar fasciitis at 12 months after treatment, but the average VAS scores for SW and CSI at 12 months after treatment were similar. Still, more studies comparing the long-term therapeutic effect of the 2 treatments are needed.

The earliest article included in the literature review^31^, which was published in 1996, mentioned the use of SW for plantar fasciitis treatment, and many studies^11,30,32–35^ comparing the effectiveness of SW and CSI for plantar fasciitis were published after that one. One meta-analysis^27^ suggested that setting the highest and mostly tolerable energy output within medium-intensity range (energy flux density 0.08–0.28 mJ/mm^2^) is the ideal alternative when applying focal SW therapy on plantar fasciitis. One of the 5 RCTs comparing the effectiveness of SW and CSI in the present study used low-intensity SW and 4 used medium-intensity SW in their SW groups. Of the 4 RCTs comparing SW and CSI at 3 months, all used medium-intensity SW in their SW groups. Thus, it seems that even in using the most effective SW therapeutic range, pain reduction in SW was not greater than that observed in CSI.

Previous studies have discussed the efficacy of orthosis, including insole^8,36^ or splinting^37,38^, for plantar fasciitis treatment. However, only one study, by Yucel *et al*.^8^, has compared the therapeutic effect of insole use and CSI, and it showed that pain reduction was better in the CSI group than in the insole group 1 month after treatment. However, head-to-head comparison studies between orthosis and CSI for plantar fasciitis remain scarce. Wearing an orthosis for plantar fasciitis treatment usually takes long treatment period. Currently, no standard guideline exists on how many times a day and for how many days an orthosis should be worn to achieve the desired therapeutic effect in treating plantar fasciitis. The major problem that researchers encounter when they study this topic is that of measuring patient compliance.

Although oral NSAIDs are frequently prescribed to patients with acute or chronic musculoskeletal or soft tissue pain, current evidence does not support its efficacy in treating plantar fasciitis. A randomized, prospective, placebo-controlled study showed that there were no statistically significant differences between the placebo and oral NSAID groups in pain relief at 1, 2 or 6 months^39^. Another head-to-head study, by Biswas *et al*.^9^, comparing oral NSAIDs and CSI showed that pain relief was significantly greater after CSI than after oral NSAIDs, and the recurrence of heel pain was significantly higher in the oral NSAID group^9^. This study^9^ was the only trial included in the meta-analysis that compared VAS score reduction in CSI and NSAIDs. Our results also favored CSI, although it was limited to a single trial comparison. Considering the side effects of oral NSAIDs and their limited therapeutic effect, NSAIDs as a treatment option for plantar fasciitis depend on the risk and benefits evaluation done by the clinician.

Because of its known anti-inflammatory effects, RT has been used for >60 years. However, its exact mechanism remains unknown^10^. Head-to-head comparison studies between RT and CSI for plantar fasciitis are also rare. In a study by Canyilmaz *et al*.^10^, patients with plantar fasciitis randomly received either RT (a total dose of 6.0 Gy given as 1 Gy 3 times a week for 2 weeks) or CSI (1 injection with 1 mL of 40 mg methylprednisolone and 0.5 mL of 1% lidocaine), and the results in the RT group were significantly superior to those in the CSI group (VAS, *P* < 0.001) at 3 and 6 months after treatment. Although evidence suggests that the risk of cancer following low to intermediate RT for benign disease is small, caution must be exercised when considering RT use in younger adults^40^. Niewald *et al*.^41^ enrolled 127 patients to receive either a standard dose or a very low dose of RT for plantar fasciitis, in which 9 patients (7%) had to be excluded after randomization because they withdrew their informed consent to radiotherapy. Obtaining patients’ consent to RT in treating plantar fasciitis is still challenging.

Of the 9 RCTs, only a few articles mentioned the complications after treatments. Porter *et al*.^30^ and Yucel *et al*.^35^ reported that 8 and 4 patients, respectively, required analgesia due to pain after CSI. In the study by Biswas *et al*.^9^, 60 patients received CSI, 6 reported injection site erythema, 2 reported injection site infection and 2 reported plantar fascia rupture. In total, 316 patients in the 9 RCTs received CSI, of which 2 patients (0.6%) reported plantar fascia rupture and 2 (0.6%) reported injection site infection. CSI provides some relief from pain, but can be associated with severe complications. A retrospective chart review showed that, after an average of 2.67 blind injections of steroid, 2.4% of patients with plantar fasciitis (3/123) experienced plantar fascia rupture^42^. However, some non-invasive treatments would also result in side effects or complications. In a study by Porter *et al*.^30^, 6 patients reported throbbing pain and erythema and 4 reported severe headache or migraine after SW. Yucel *et al*.^35^ also reported that 2 patients had a mild throbbing sensation that lasted for an average of 5 days and another 2 had mild erythema after SW. Biswas *et al*.^9^ reported that 40 patients had gastritis, 5 had esophagitis, 8 had pruritus and 5 experienced bloating after oral NSAIDs.

There are some limitations to our meta-analysis. First, due to the limited number of trials, not all non-invasive treatment options could be included. Therefore, the non-invasive treatments covered in the present study may not represent all types of non-invasive treatments, and the results of comparing CSI with certain non-invasive treatments other than SW, PT, insole and NSAIDs could not be obtained. Second, outcome measurements, other than VAS, were not universally used in all included trials. Without these data, we could not discern the comprehensive outcome of various treatment options in patients with plantar fasciitis. Third, because the included trials lack long-term data, we could provide only short-term (i.e. up to 3 months) comparison data. Despite these limitations, our study demonstrates a clear comparison of therapeutic outcome between CSI and non-invasive treatments for plantar fasciitis and provides important information to clinicians that could be useful in the selection of therapeutic options.

In conclusion, compared with non-invasive treatments, CSI treatment for plantar fasciitis is associated with short-term (i.e. 3 months) pain reduction. CSI is superior to PT in relieving pain at 1.5 months. CSI and SW have similar probabilities of providing pain relief within 3 months of follow-up. Large-scale, well-designed RCTs comparing different non-invasive treatment options with CSI, with various objective outcome assessments and longer follow-up, are required in the future.
